# Oncogenic RAS promotes MYC protein stability by upregulating the expression of the inhibitor of apoptosis protein family member Survivin

**DOI:** 10.1016/j.jbc.2022.102842

**Published:** 2022-12-26

**Authors:** Wen-Hsuan Chang, Yinzhe Liu, Emma A. Hammes, Kirsten L. Bryant, Richard A. Cerione, Marc A. Antonyak

**Affiliations:** 1 Department of Chemistry and Chemical Biology, Cornell University, Ithaca, New York, USA; 2 Lineberger Comprehensive Cancer Center, University of North Carolina at Chapel Hill, Chapel Hill, North Carolina, USA; 3 Department of Pharmacology, University of North Carolina at Chapel Hill, Chapel Hill, North Carolina, USA; 4 Department of Molecular Medicine, Cornell University, Ithaca, New York 14853, USA

**Keywords:** pancreatic cancer, KRAS, survivin, MYC, CIP2A, autophagy, ERK1/2, extracellular signal–regulated kinase 1/2, MEF, mouse embryonic fibroblast, PDAC, pancreatic ductal adenocarcinoma, PP2A, protein phosphatase 2A, qPCR, quantitative PCR

## Abstract

The small GTPase KRAS is frequently mutated in pancreatic cancer and its cooperation with the transcription factor MYC is essential for malignant transformation. The key to oncogenic KRAS and MYC working together is the stabilization of MYC expression due to KRAS activating the extracellular signal–regulated kinase 1/2, which phosphorylates MYC at serine 62 (Ser 62). This prevents the proteasomal degradation of MYC while enhancing its transcriptional activity. Here, we identify how this essential signaling connection between oncogenic KRAS and MYC expression is mediated by the inhibitor of apoptosis protein family member Survivin. This discovery stemmed from our finding that Survivin expression is downregulated upon treatment of pancreatic cancer cells with the KRAS^G12C^ inhibitor Sotorasib. We went on to show that oncogenic KRAS increases Survivin expression by activating extracellular signal–regulated kinase 1/2 in pancreatic cancer cells and that treating the cells either with siRNAs targeting Survivin or with YM155, a small molecule that potently blocks Survivin expression, downregulates MYC and strongly inhibited their growth. We further determined that Survivin protects MYC from degradation by blocking autophagy, which then prevents cellular inhibitor of protein phosphatase 2A from undergoing autophagic degradation. Cellular inhibitor of protein phosphatase 2A, by inhibiting protein phosphatase 2A, helps to maintain MYC phosphorylation at Ser 62, thereby ensuring its cooperation with oncogenic KRAS in driving cancer progression. Overall, these findings highlight a novel role for Survivin in mediating the cooperative actions of KRAS and MYC during malignant transformation and raise the possibility that targeting Survivin may offer therapeutic benefits against KRAS-driven cancers.

The RAS family of small GTPases, including KRAS, HRAS, and NRAS, function as molecular switches that are tightly controlled and transduce signals between cell surface receptor tyrosine kinases and effectors that regulate several cellular processes, including cell growth, migration, attachment, differentiation, and survival (1, 2). However, the different RAS isoforms are also frequently mutated in cancer, resulting in their constitutive activation. For example, KRAS is mutated in nearly 95% of all pancreatic ductal adenocarcinoma (PDAC) patients, a particularly aggressive and deadly form of cancer (2, 3), where it has been shown to potently activate extracellular signal–regulated kinase 1/2 (ERK1/2) and PI3K and give rise to enhanced cell growth, chemoresistance, migration, and invasion, as well as metastatic spread. Treatment of PDAC cells with recently developed inhibitors that target specific oncogenic KRAS mutants, that is, Sotorasib, Adagrasib, and MRTX1133, or block different components of the ERK1/2 or PI3K pathways (4, 5, 6), cause the cells to lose their aggressive phenotypes, underscoring the importance of RAS signaling in cancer progression (7, 8, 9). However, PDAC patients often become resistant to therapies, highlighting the continued need to better understand how KRAS mediates its oncogenic effects.

In order for RAS to transform primary cells, it needs to cooperate with another proto-oncogene, often the transcription factor MYC (9, 10, 11), as originally demonstrated in a landmark study, where only after coexpressing oncogenic HRAS with MYC in primary fibroblasts did the cells exhibit the characteristics of cancer cells, that is, loss of contact inhibition, anchorage-independent growth, and tumor formation in mice (12). The importance of MYC in RAS-induced cellular transformation was further reinforced by studies showing that oncogenic RAS increases MYC protein expression levels by preventing its degradation (9, 12). A salient feature of this regulation involves the phosphorylation state of MYC (13). The activation of ERK1/2 by KRAS results in the phosphorylation of MYC at Ser 62, which stabilizes the cellular expression of MYC and enhances its transcriptional activity (14, 15, 16). In cells that have not undergone oncogenic transformation, MYC is subsequently phosphorylated on threonine 58 (Thr 58) by glycogen synthase kinase 3β, which then helps to recruit the protein phosphatase 2A (PP2A), resulting in the dephosphorylation of Ser 62 and thereby targeting MYC for degradation in the proteasome (15, 17, 18). However, in cancer cells expressing oncogenic forms of KRAS, the balance between the phosphorylation-dephosphorylation states of MYC is shifted toward an increased phosphorylation at Ser 62, thus reducing the amount MYC that undergoes ubiquitination and degradation. Here, we show that an essential component of the signaling pathway by which oncogenic KRAS enhances MYC expression is the protein Survivin, which is a member of the inhibitor of apoptosis protein family.

Survivin is expressed at very low levels in most adult tissues (19, 20); however, its expression is significantly upregulated in a number of cancers, where it is best known for promoting therapy resistance by inhibiting the activation of caspases (19, 21, 22). Survivin has also been reported to influence cell division, migration, as well as inhibit autophagy, a process by which cellular components are targeted for degradation in lysosomes (19, 23, 24, 25, 26, 27, 28). In this report, we now identify a novel role for Survivin in the ability of oncogenic KRAS to drive malignant transformation. We first describe how Survivin expression is significantly increased in PDAC cells and RAS-transformed cells (22, 29) and that its depletion using siRNAs or by treatment with YM155, a small molecule that potently inhibits Survivin transcription, blocks their growth (30, 31). We then elucidate the intricate regulatory mechanism by which Survivin mediates the effects of oncogenic KRAS on the phosphorylation status and cellular stability of MYC, through its ability to block the autophagic degradation of CIP2A, a protein that binds and protects MYC from dephosphorylation by PP2A, thus ensuring the sustained phosphorylation of MYC at Ser 62 (32, 33, 34). This then explains how oncogenic KRAS, by signaling the upregulation of Survivin expression, enhances the cellular expression and transcriptional activity of MYC, thereby setting the stage for oncogenic KRAS and MYC to work together to drive oncogenic transformation.

## Results

### Survivin is important for the growth and viability of oncogenic KRAS-dependent cancer cells

**Figure 1:**
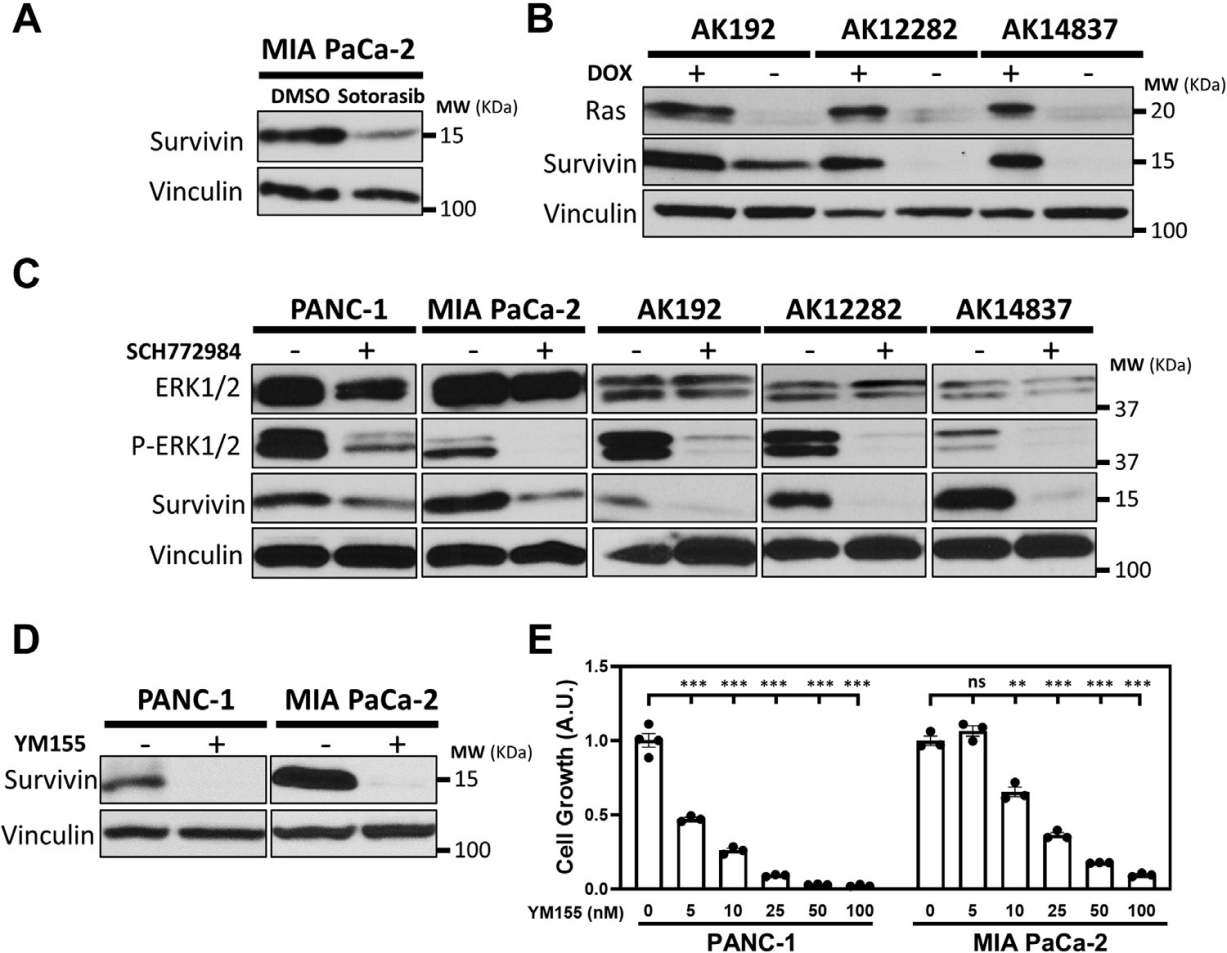
**RAS-ERK signaling promotes Survivin expression.***A*, representative Western blot analysis of Survivin expression in MIA PaCa-2 cells treated without (DMSO) or with 1 μM Sotorasib. *B*, representative Western blot analysis of KRAS and Survivin expression in AK192, AK12282, and AK14837 cells expressing a doxycycline (DOX)-inducible form of KRAS^G12D^. *C*, representative Western blot analysis of ERK1/2, phospho-ERK1/2 (P-ERK1/2), and Survivin expression in AK192, AK12282, and AK14837 cells expressing KRAS^G12D^, as well as in PANC-1 and MIA PaCa-2 cells, treated without or with 1 μM SCH772984. *D*, representative Western blot analysis of Survivin expression in PANC-1 and MIA PaCa-2 cells treated without or with 250 nM YM155 for 24 h. *E*, cell counting kit-8 (CCK-8) cell growth assays were performed on PANC-1 and MIA PaCa-2 cells treated with increasing concentrations of YM155. In (*A*–*D*), vinculin was used as the loading control. All experiments involving Western blots were performed a minimum of three independent times, with similar results being obtained each time. In (*E*), data are means ± standard errors of at least three experiments; ∗∗*p* < 0.01, ∗∗∗*p* < 0.001, and nonsignificant (ns) by Student’s *t* test. ERK1/2, extracellular signal–regulated kinase 1/2.

To better understand how oncogenic KRAS promotes malignant transformation, we determined the effects of treating human MIA PaCa-2 PDAC cells with Sotorasib (AMG-510), which specifically and irreversibly inhibits the oncogenic KRAS^G12C^ mutant. The growth of the cells was highly sensitive to treatment with the inhibitor (Fig. S1). We also determined that the expression of the cell growth and survival protein Survivin was strongly downregulated under these same conditions (Fig. 1*A*, top panel), suggesting that Survivin plays an important role in the ability of oncogenic KRAS to transform cells.

We recently showed that when the oncogenic KRAS^G12D^ mutant, commonly found in PDAC, was ectopically expressed in mouse embryonic fibroblasts (MEFs), it strongly upregulated Survivin expression (22). We then examined whether this was also the case in mouse PDAC cell lines engineered to express an inducible form of KRAS^G12D^. When this oncogenic KRAS mutant was inducibly expressed upon treatment of AK192, AK12282, and AK14837 cells with doxycycline (Fig. 1*B*, top panel), the relative induction of Survivin expression nicely correlated with KRAS^G12D^ expression (Fig. 1*B*, second panel).

Since the ability of oncogenic RAS to activate ERK alters gene transcription and activates proteins that increase cell growth and survival (2), we determined whether ERK activation was required for the increased Survivin expression observed in PDAC cells expressing mutant forms of KRAS. AK192, AK12282, and AK14837 cells induced to express KRAS^G12D^, as well as PANC-1 and MIA PaCa-2 cell lines, were treated with the ERK1/2 inhibitor SCH772984 for 24 h. While ERK1/2 expression varied between the different cell lines, the amount of protein detected within each cell line was not affected by treatment with the inhibitor, with the exception of PANC-1 cells, which showed a modest reduction in ERK1/2 expression (Fig. 1*C*, top panel). However, ERK1/2 activation in the cells was effectively blocked by SCH772984, as determined using an antibody that recognizes the phosphorylated, activated form of ERK1/2 (Fig. 1*C*, second panel). Under conditions where ERK1/2 activation was inhibited, there was a corresponding reduction in Survivin expression (Fig. 1*C*, third panel).

To investigate the importance of Survivin in RAS-driven cancer cell lines, we took advantage of YM155, which inhibits Survivin expression by blocking the transcription factors Sp1 and interleukin enhancer-binding factor 3 (35, 36). Treatment of PANC-1 and MIA PaCa-2 cells with YM155 for 8 h caused a striking reduction in Survivin expression (Fig. 1*D*, top panel). When these cell lines were treated with increasing concentrations of YM155, a dose-dependent reduction in cell growth was observed (Fig. 1*E*).

### Survivin enhances MYC expression levels

**Figure 2:**
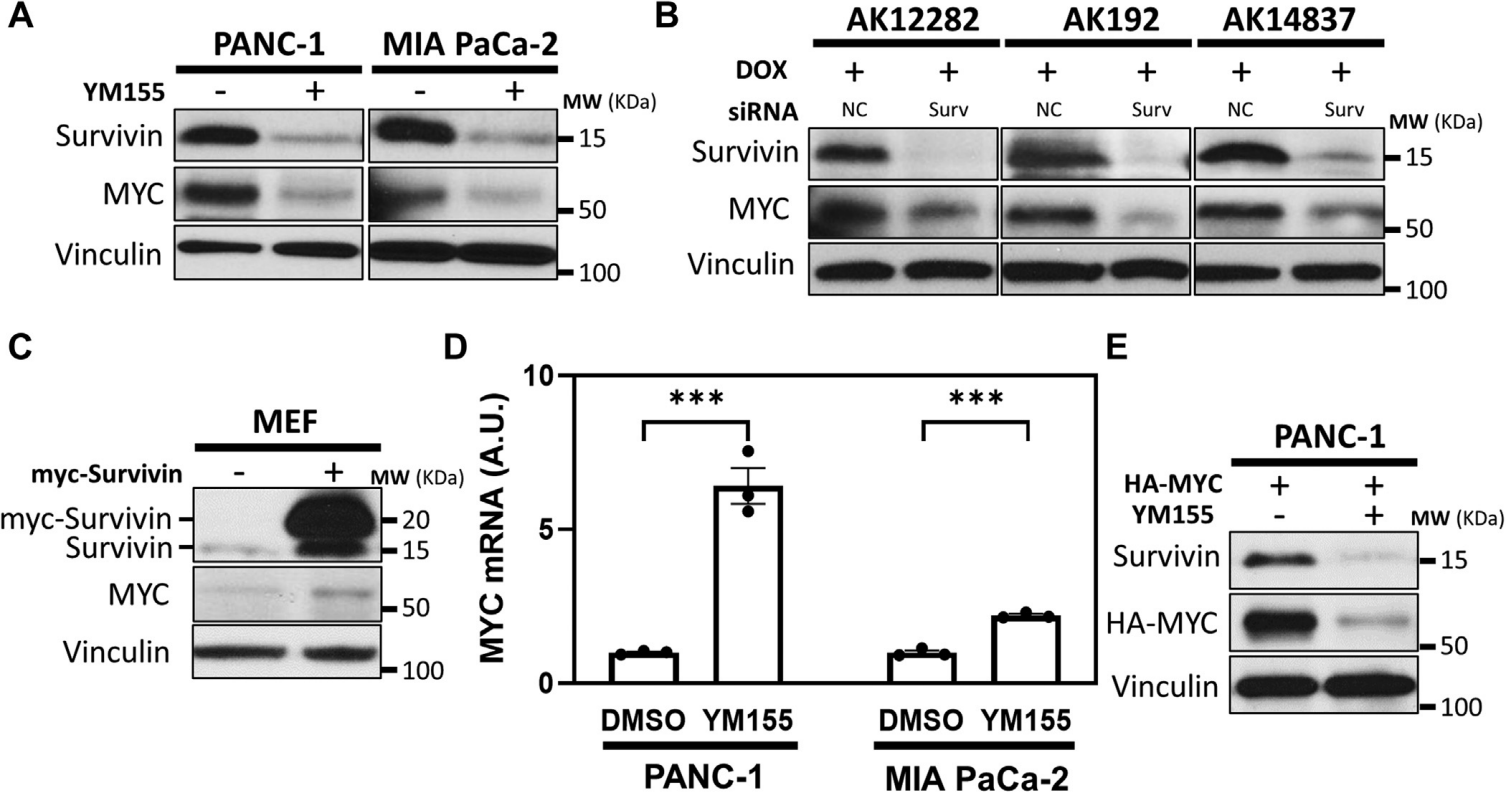
**Survivin regulates MYC expression.***A*, representative Western blot analysis of Survivin and MYC expression in PANC-1 and MIA PaCa-2 cells treated without or with 250 nM YM155. *B*, representative Western blot analysis of Survivin and MYC expression in AK192, AK12282, and AK14837 cells induced to express KRAS^G12D^ and transfected with negative control (NC) or Survivin-targeting (Surv) siRNAs. *C*, representative Western blot analysis of Survivin and MYC expression in MEFs ectopically expressing the vector alone or Myc-tagged Survivin (myc-Survivin). *D*, quantitative PCR (qPCR) analysis of MYC mRNA levels, relative to actin mRNA levels, in PANC-1 and MIA PaCa-2 cells treated without (DMSO) or with 250 nM YM155. *E*, representative Western blot analysis of Survivin and HA-MYC expression in PANC-1 cells ectopically expressing HA-tagged MYC and treated with 250 nM YM155 for 24 h. In (*A*–*C and E*), vinculin was used as the loading control. All experiments involving Western blots were performed a minimum of three independent times, with similar results being obtained each time. In (*D*), data are means ± standard errors of at least three experiments; ∗∗∗*p* < 0.001 by Student’s *t* test. MEF, mouse embryonic fibroblast.

To gain further insights into the actions of Survivin, we determined whether depleting PDAC cells of Survivin affected the expression of proteins with known roles in promoting RAS-induced cellular transformation. One classic example is the transcription factor and proto-oncogene MYC, which is upregulated in RAS-dependent cancers, where it has been shown to promote different aspects of cancer progression. Treating PANC-1 and MIA PaCa-2 cells with either YM155 (Fig. 2*A*, top panels) or two different siRNAs that target Survivin (Surv #1 and Surv #2; Fig. S2*A*, top panels) caused a marked decrease in the levels of MYC (Fig. 2*A* and Fig. S2*A*, second panels). Similar reductions in MYC expression also occurred when AK192, AK12282, and AK14837 cells expressing KRAS^G12D^ were transfected with an siRNA-targeting Survivin (Fig. 2*B*, top two panels). Moreover, ectopic expression of Survivin in MEFs (Fig. 2*C*, top panel) was sufficient to increase MYC levels (Fig. 2*C*, second panel).

These findings raised an interesting question: how does downregulation of Survivin expression cause MYC levels to decrease? Based on previous work that suggested Survivin can alter gene expression (37, 38, 39), we initially suspected that this effect was due to a reduction in the transcription of the MYC gene. However, this is not the case, as quantitative PCR (qPCR) performed on RNA isolated from YM155-treated PANC-1 and MIA PaCa-2 cells showed a significant increase in MYC transcript levels, compared to DMSO-treated control cells (Fig. 2*D*). Thus, it appeared that upon depletion of Survivin, cells compensate for the corresponding reduction in MYC protein expression by upregulating its transcription. We then considered whether Survivin might influence the stability of the MYC protein. Cells transfected with HA-tagged MYC (HA-MYC) were either left untreated or were treated with YM155, and the resulting expression levels of HA-MYC were determined for each condition. Compared to the relatively high expression of HA-MYC in untreated cells, considerably less expression of HA-MYC was detected in YM155-treated cells (Fig. 2*E*). The fact that depleting Survivin downregulated both endogenous and ectopically expressed MYC indicated that Survivin enhances MYC expression by maintaining its stability.

**Figure 3:**
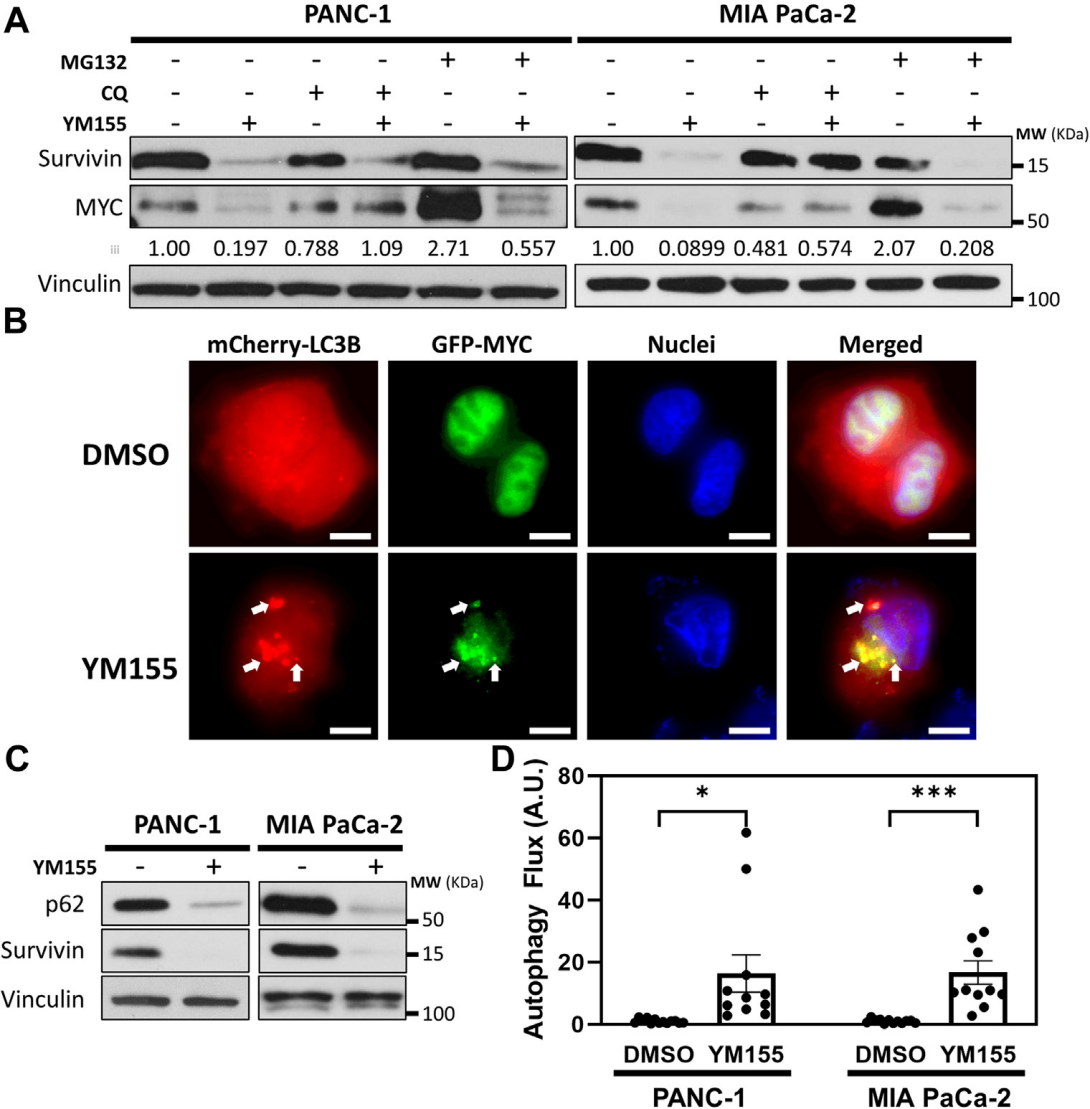
**Survivin stabilizes the cellular expression of MYC by inhibiting autophagy.***A*, representative Western blot analysis of Survivin and MYC expression in PANC-1 and MIA PaCa-2 cells treated with the indicated combinations of 1 μM MG-132, 25 μM chloroquine (CQ), and 250 nM YM155. The relative changes in MYC expression were quantified using densitometry and listed below the corresponding blots. *B*, representative fluorescent microscopy images of PANC-1 cells ectopically expressing mCherry-LC3B and GFP-MYC and treated without (DMSO) or with 250 nM YM155 for 24 h. The cells were also stained with Hoechst stain to label nuclei. Arrows indicate areas of mCherry-LC3B and GFP-MYC colocalization. The scale bars represent 10 μm, and similar results were obtained in three separate experiments. *C*, representative Western blot analysis of p62 and Survivin expression in PANC-1 and MIA PaCa-2 cells treated without or with 250 nM YM155. *D*, autophagic flux was determined by performing live-cell confocal microscopy (see Fig. S3*B*) on PANC-1 and MIA PaCa-2 cells ectopically expressing mCherry-EGFP-LC3B and treated without (DMSO) or with 250 nM YM155 for 24 h. In (*A and C*), vinculin was used as the loading control. All experiments involving Western blots and immunofluorescence were performed a minimum of three independent times, with similar results being obtained each time. In (*D*), data are means ± standard errors of at least three experiments; ∗*p* < 0.05 and ∗∗∗*p* < 0.001 by Student’s *t* test.

We next inhibited some of the major degradation machinery in cells, specifically, proteasomes and autophagosomes/lysosomes, in order to determine whether this ablates the effects of depleting Survivin on MYC expression. Treatment of PANC-1 and MIA PaCa-2 cells with the proteasome inhibitor MG-132 caused a significant increase in MYC expression (Fig. 3*A*, second panel, compare lanes 1 and 5 for each cell line), consistent with previous studies showing that MYC can be degraded in proteasomes (15, 16, 17, 18). However, we found that the increase in MYC expression caused by treating cells with MG-132 was reversed by YM155 treatment (Fig. 3*A*, second panel, compare lanes 5 and 6 for each cell line), thus indicating that the ability of Survivin to help sustain MYC expression was not attributable to a prevention of proteasomal degradation. We then determined whether inhibiting autophagy-dependent lysosomal activity using chloroquine would affect the ability of YM155 to reduce MYC expression. The amount of MYC detected in PANC-1 and MIA PaCa-2 cells treated with chloroquine alone, or chloroquine and YM155, were nearly identical (Fig. 3*A*, second panel, compare lanes 3 and 4 for each cell line). The reduction in MYC expression caused by the siRNA-mediated knockdown of Survivin was also blocked by treatment with chloroquine (Fig. S2*B*), consistent with the idea that Survivin plays an important role in preventing autophagy/lysosomal-mediated degradation of MYC. Note, Survivin expression in MIA PaCa-2 cells treated with YM155 and chloroquine is higher, compared to when these cells with YM155 alone. This suggests that in these cancer cells, the cellular lifetime of Survivin is of sufficient duration such that its ability to inhibit autophagic degradation enables its expression to be maintained.

To further confirm a role for Survivin in blocking autophagy and thereby helping to sustain MYC expression, three additional experiments were performed. In one set of experiments, cells transfected with GFP-tagged MYC (GFP-MYC) and an mCherry-tagged version of the autophagosome marker microtubule-associated protein light chain 3B (mCherry-LC3B) were either left untreated or treated with YM155 for 8 h, before being visualized by fluorescent microscopy. In the untreated cells, GFP-MYC was predominantly nuclear, while mCherry-LC3B was diffusely expressed throughout the cytosol (Fig. 3*B*, top panels). However, YM155 treatment of the cells caused GFP-MYC and mCherry-LC3B to localize together in the cytosol in structures that resemble autophagosomes (Fig. 3*B*, bottom panels, arrows). Cells cultured under these same conditions were also assayed for apoptosis, as readout by the cleavage of caspase 3. Fig. S3*A* shows that very little cleaved caspase 3 could be detected in these cells.

In the second set of experiments, lysates collected from PANC-1 and MIA PaCa-2 cells treated with YM155 were analyzed for changes in the expression of the autophagy-related protein p62, also referred to as sequestosome 1 (SQSTM1). Generally, p62 accumulates in cells under conditions when autophagy is limited, while its expression decreases when the rates of autophagy are increased. Depleting cells of Survivin *via* treatment with YM155 markedly downregulated the expression of p62 (Fig. 3*C*, top panel).

In the third set of experiments, autophagic flux in PANC-1 and MIA PaCa-2 cells depleted of Survivin was determined by monitoring changes (i.e., the flux) in the fluorescence of the ectopically expressed tandem-tagged reporter construct mCherry-EGFP-LC3B, which labels autophagosomes and can be visualized using live-cell confocal microscopy (Fig. S3*B*). This assay takes advantage of the fact that EGFP fluorescence (but not mCherry fluorescence) is highly sensitive to changes in pH, which occurs when autophagosomes fuse with lysosomes. The acidic environment of lysosomes causes a loss in EGFP fluorescence, which is used to determine autophagic flux. Fig. 3*D* shows that treatment of either cell line with YM155 caused a robust, ∼16-fold increase in autophagic flux. Similar results were obtained when Survivin was depleted from the cells using siRNAs (Fig. S3, *C* and *D*). Collectively, these findings strongly suggest that Survivin maintains the stability of MYC by preventing autophagy/lysosomal-mediated degradation.

### Survivin, by influencing the phosphorylation status of MYC, protects it from degradation

**Figure 4:**
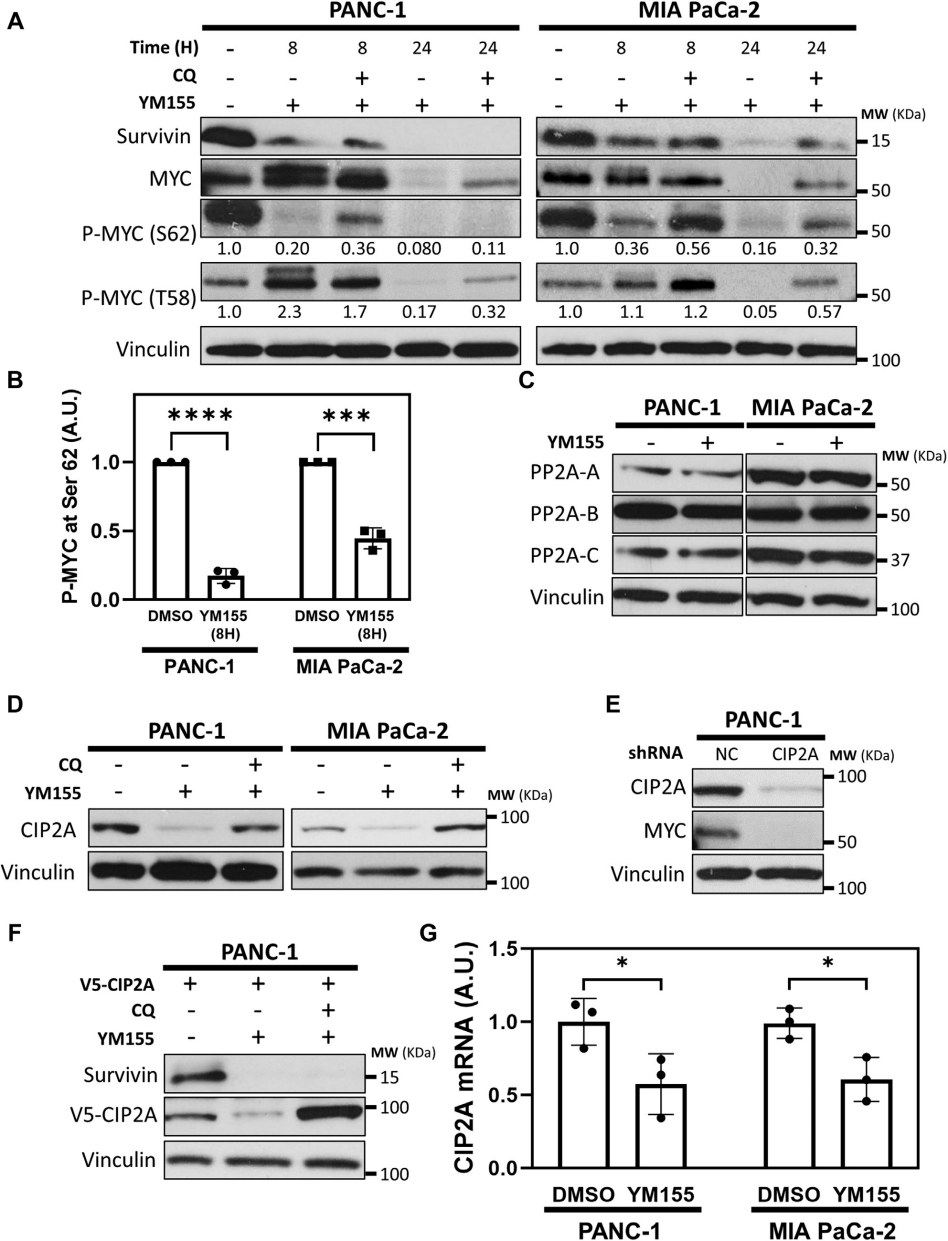
**Survivin maintains the phosphorylation of Ser 62 in MYC.***A*, representative Western blot analysis of Survivin, MYC, phospho-MYC (P-MYC) Ser 62, and P-MYC Thr 58 levels in PANC-1 and MIA PaCa-2 cells treated with the indicated combinations of 25 μM chloroquine (CQ) and 250 nM YM155 for either 8 or 24 h. The relative changes in MYC phosphorylation at Ser 62 and Thr 58 were quantified using densitometry and listed below the corresponding blots. *B*, quantification of the relative changes in the phosphorylation of MYC at Ser 62 in cells treated without (DMSO) or with YM155 for 8 h. *C*, representative Western blot analysis of PP2A-A, PP2A-B, and PP2A-C expression in PANC-1 and MIA PaCa-2 cells treated without (DMSO) or with 250 nM YM155. *D*, representative Western blot analysis of Survivin and CIP2A expression in PANC-1 and MIA PaCa-2 cells treated with the indicated combinations of 25 μM chloroquine (CQ) and 250 nM YM155. *E*, representative Western blot analysis of CIP2A and MYC expression in PANC-1 cells expressing negative control (NC) and CIP2A-targeting shRNAs. *F*, representative Western blot analysis of Survivin and V5-tagged CIP2A expression in PANC-1 cells ectopically expressing V5-tagged CIP2A and treated with the indicated combinations of 25 μM chloroquine (CQ) and 250 nM YM155. *G*, quantitative PCR (qPCR) analysis of CIP2A mRNA levels, relative to actin mRNA levels, in PANC-1 and MIA PaCa-2 cells treated without (DMSO) or with 250 nM YM155 for 24 h. In (*A, C*–*F*), vinculin was used as the loading controls. All experiments involving Western blots were performed a minimum of three independent times, with similar results being obtained each time. In (*B and F*), data are means ± standard errors of at least three experiments; ∗*p* < 0.05, ∗∗∗*p*< 0.001, and ∗∗∗∗*p* < 0.0001 by Student’s *t* test. PP2A, protein phosphatase 2A.

It is well established that the stability of MYC is tightly controlled by its phosphorylation status. When phosphorylated on Ser 62, MYC is protected from degradation. However, phosphorylation of Thr 58 results in the dephosphorylation of Ser 62, targeting MYC for degradation (15). Therefore, we set out to determine whether Survivin maintains the stability of MYC by influencing its phosphorylation state. PANC-1 and MIA PaCa-2 cells were treated with a combination of YM155 and chloroquine for either 8 or 24 h and then were subjected to Western blot analysis. Although MYC expression in these cells was strongly downregulated following 24 h of YM155 treatment (Fig. 4*A*, second panel, compare lanes 1 and 4 for each cell line), its expression was largely unaffected by an 8-h treatment with the drug, even though Survivin expression was inhibited (Fig. 4*A*, top two panels, compare lanes 1 and 2 for each cell line). Under these shorter time treatments with YM155, we found that the level of MYC phosphorylation at Ser 62 was markedly decreased, while phosphorylation of Thr 58 was unchanged (Fig. 4*A*, third and fourth panels, compare lanes 1 and 2 for each cell line and 4B). The effect that YM155 has on the phosphorylation of MYC at Ser 62 cannot be explained by a loss of ERK1/2 activity, since treating these cells with YM155, or transfecting them with Survivin-targeting siRNAs, was found to increase ERK1/2 phosphorylation (Fig. S4, *A* and *B*). Moreover, while treating the cells with chloroquine together with YM155 for 24 h helped to maintain a low level of MYC expression, compared to cells treated with YM155 alone, phosphorylation at Ser 62 remained low, thus indicating that Survivin also plays a role in maintaining the phosphorylation of a key residue that regulates the cellular half-life of MYC.

Since dephosphorylation of Ser 62 in MYC is mediated by PP2A (15), we examined whether its expression was affected by depleting cells of Survivin. PP2A is composed of three different subunits, PP2A-A, PP2A-B, and PP2A-C (18, 40), and the expression of each subunit was similar in untreated cells and in cells treated with YM155 (Fig. 4*C*). However, we found that CIP2A, a protein that is overexpressed in cancers and shown to bind MYC and prevent PP2A from dephosphorylating Ser 62^33^, was robustly downregulated upon treatment with either YM155 (Fig. 4*D*, top panels, compare lanes 1 and 2 for each cell line) or siRNAs that target Survivin (Fig. S4*C*). Knocking down CIP2A expression resulted in a decrease in MYC levels that was comparable to the reduction seen when cells were depleted of Survivin using YM155 or siRNA (Fig. 4*E*).

We then set out to determine whether Survivin influences CIP2A expression by inhibiting autophagy/lysosomal activity. Since treatment of PDAC cells with YM155 increases autophagy and downregulates CIP2A expression, we examined how the levels of CIP2A were affected when cells depleted of Survivin were treated with the autophagy/lysosomal inhibitor chloroquine. As shown in Fig. 4*D* (top panel, compare lanes 2 and 3 for each cell line) and Fig. S4*D*, treatment of PANC-1 and MIA PaCa-2 cells with chloroquine was able to fully reverse the inhibitory effects of YM155 and Survivin-targeting siRNA treatments on CIP2A expression. Similarly, we found that treating cells ectopically expressing a V5-tagged form of CIP2A (V5-CIP2A) with YM155 severely compromise its expression, while chloroquine treatment blocked this effect (Fig. 4*F*, second panel, compare lanes 2 and 3). Moreover, qPCR performed on RNA collected from PANC-1 and MIA PaCa-2 cells depleted of Survivin showed a significant reduction in CIP2A transcript, compared to untreated cells (Fig. 4*G*). These findings suggest that Survivin upregulates the expression of CIP2A by increasing its transcription as well as by protecting the protein from autophagy/lysosomal-dependent degradation.

### Downregulating MYC is important for YM155-induced PDAC cell growth inhibition

**Figure 5:**
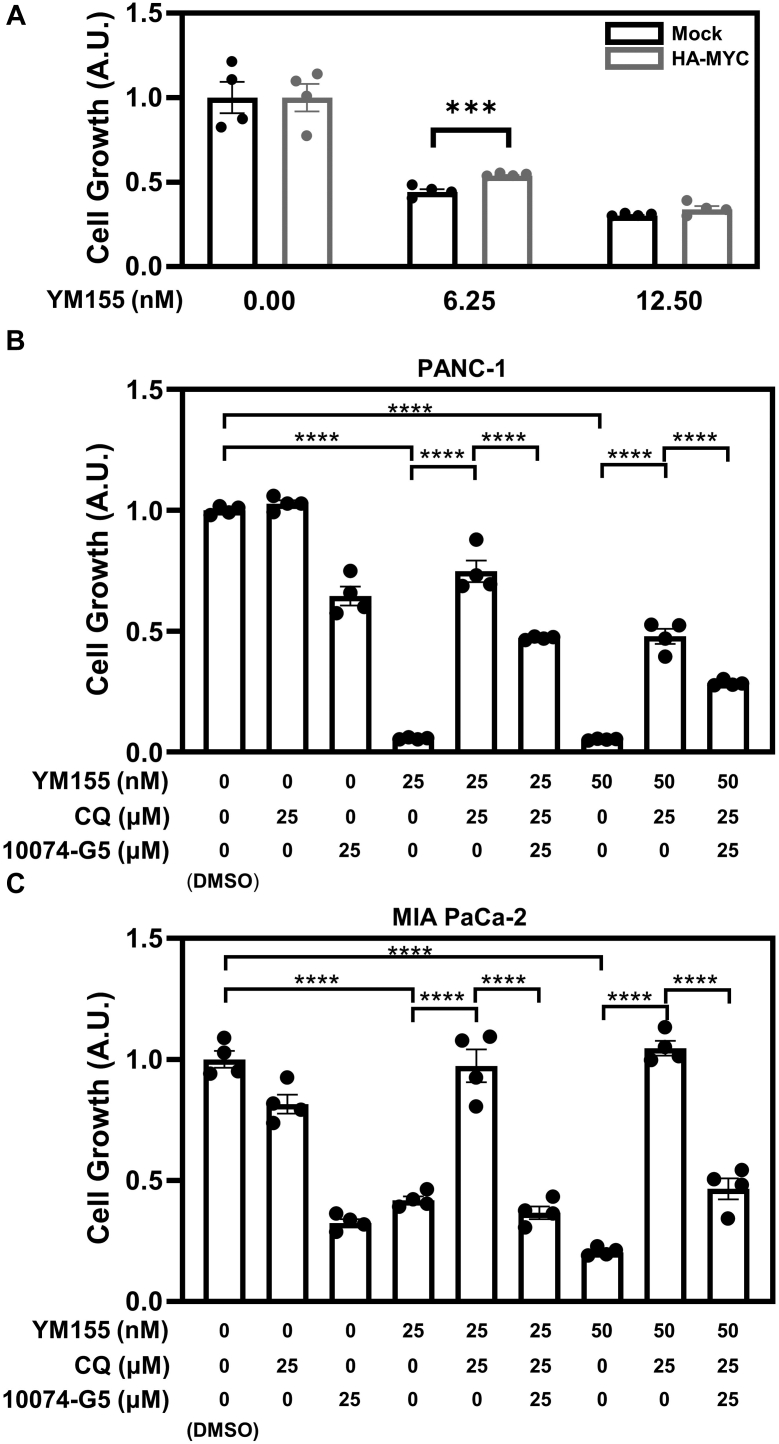
**Downregulating MYC expression is important for the cell growth inhibition caused by YM155 treatment.***A*, CCK-8 cell growth assays were performed on PANC-1 cells ectopically expressing either the vector alone (Mock) or HA-tagged MYC and treated without (DMSO) or with the indicated concentrations of YM155. *B*-*C*, CCK-8 cell growth assays were performed on PANC-1 (*B*) and MIA PaCa-2 (*C*) cells treated with the indicated concentrations and combinations of YM155, chloroquine (CQ), and 10074-G5. In (*A*–*C*), data are means ± standard errors of at least three experiments; ∗∗∗*p* < 0.001, and ∗∗∗∗*p* < 0.0001 by Student’s *t* test.

We have shown that depleting PDAC cells of Survivin significantly inhibited their growth. Since MYC levels are also decreased under this same condition, we asked whether depleting cells of Survivin using YM155 could be reversed by increasing the expression of MYC. However, ectopically expressing MYC in cells treated with YM155 resulted in its rapid degradation (see Fig. 2*F*) and thus only minimally increased cell growth (Fig. 5*A*). As an alternative approach, we performed growth assays on cells treated with both YM155 and chloroquine, such that Survivin expression was inhibited while MYC levels were maintained. Treating PANC-1 cells with 25 nM or 50 nM of YM155 was shown to inhibit their growth by 95 % (Fig. 5*B*), whereas treating the cells with either 25 nM or 50 nM of YM155 and chloroquine restored their growth to 75 % and 48 %, respectively, compared to control cells. Similar results were obtained with MIA PaCa-2 cells (Fig. 5*C*).

To further confirm the importance of downregulating MYC for YM155-induced cell growth inhibition, we used the small molecule inhibitor 10074-G5, which interferes with the ability of MYC to function as a transcription factor by disrupting the formation of MYC-MAX dimers (41). Treating PANC-1 cells and MIA PaCa-2 cells with 10074-G5 was sufficient to decrease their growth by at least 60 % (Fig. 5, *B* and *C*). Moreover, the ability of chloroquine to rescue the growth inhibitory effects caused by the treatment of PANC-1 and MIA PaCa-2 cells with YM155 was prevented when the cells were also treated with 10074-G5, suggesting that PDAC cells are highly dependent on Survivin to maintain MYC expression and ensure their growth.

## Discussion

KRAS is mutated in a vast majority of PDAC cases, and studies involving animal models and patient samples of this disease have established it as the principal driver of PDAC initiation and progression (1, 2). This has led to a good deal of effort being directed towards developing strategies to target KRAS, or its effectors, as a cancer treatment. Inhibitors that bind and inactivate specific oncogenic KRAS mutants are among some of the most recent and exciting developments in the field; one example being Sotorasib which targets KRAS^G12C^ and is FDA approved (7). These inhibitors have shown promise in treating different types of cancer, but like nearly all other therapeutic drug candidates that have been used to inhibit KRAS, or effectors of KRAS (i.e., the RAF-MEK-ERK1/2 and PI3K-AKT signaling pathways), acquired resistance continues to be a major problem (42, 43). Thus, to gain a better understanding of the mechanisms by which oncogenic forms of KRAS drive cancer progression, with the hope that this might highlight new therapeutic strategies for PDAC, we treated MIA PaCa-2 PDAC cells with Sotorasib and found that the normally high expression level of Survivin in these cells was strongly downregulated. This prompted us to examine the importance of Survivin in PDAC, which led to our discovery of a novel mechanism by which Survivin couples oncogenic KRAS-signaling to the expression of MYC expression during PDAC progression.

**Figure 6:**
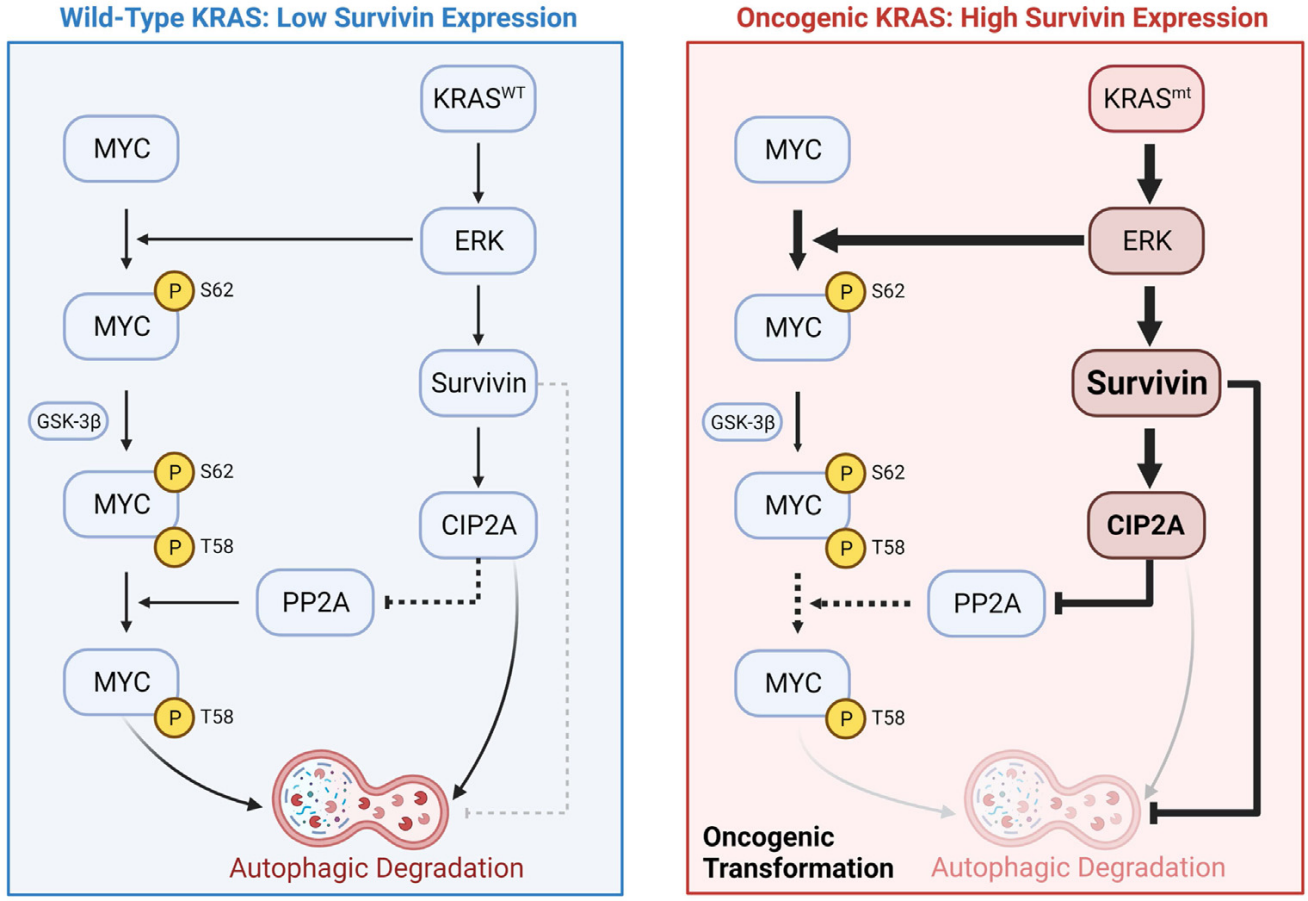
**Diagram showing how KRAS-mediated upregulation of Survivin expression stabilizes MYC to promote PDAC cell growth.** In normal cells, WT KRAS transiently activates ERK1/2, which in turn leads to the phosphorylation of Ser 62 (S62) in MYC, maintaining its stability. MYC is subsequently phosphorylated on Thr 58 (T58) by GSK-3β and dephosphorylated on Ser 62 by PP2A, which targets MYC for degradation (left side of diagram). However, mutant forms of KRAS (KRAS^mt^) potently activate ERK1/2 and increase the phosphorylation of Ser 62 in MYC, enhancing its stability. We also found that mutant KRAS-induced ERK activation strongly upregulates Survivin expression, which further enhances MYC expression by both inhibiting its autophagy-dependent degradation, as well as by promoting the expression of CIP2A, preventing PP2A from dephosphorylating Ser 62 in MYC. These effects ensure that the proper amount of MYC is expressed in PDAC cells to support their optimal growth and to work together with KRAS to drive oncogenic transformation (right side of diagram). This figure was created using BioRender.com. ERK1/2, extracellular signal–regulated kinase 1/2; GSK-3β, glycogen synthase kinase 3β; PP2A, protein phosphatase 2A.

Based on the findings described in this study, Fig. 6 provides a summary depiction of how Survivin plays a critical role in the ability of oncogenic KRAS to sustain MYC expression. It starts with oncogenic KRAS triggering the well-known protein kinase cascade that culminates in the activation of ERK1/2. This results in the upregulation of Survivin expression, as well as the phosphorylation of MYC at Ser 62, which both enhances the ability of MYC to function as a transcription factor and stabilizes its cellular expression (9, 13, 16, 43). Typically, in nontransformed healthy cells, MYC is subsequently phosphorylated on Thr 58 by glycogen synthase kinase 3β, which in turn recruits the phosphatase PP2A that catalyzes the dephosphorylation of Ser 62, targeting MYC for ubiquitination and proteasomal degradation (15). However, the upregulation of Survivin expression induced by oncogenic KRAS provides a mechanism to sustain the phosphorylation of MYC at Ser 62 and ensure that its expression is maintained. Specifically, this is due to the ability of Survivin to block autophagy and thus prevent CIP2A, an inhibitor of PP2A activity, from undergoing autophagic degradation. This finding is in line with a previous study that showed CIP2A is degraded *via* a mechanism that involves chaperone-mediated autophagy (44). By maintaining the expression of CIP2A, Survivin ensures that the phosphorylation of MYC at Ser 62 is sustained, thereby preventing MYC from being targeted to the proteasomes where it is degraded. Importantly, by blocking autophagy, Survivin also prevents MYC from being susceptible to this mechanism for degradation that would otherwise compensate for the inability of MYC to be delivered to proteasomes. Certainly, an important question for future studies concerns the specific mechanism used by Survivin to block autophagy, although recent lines of evidence suggest this may occur through its ability to interact with several autophagy-related proteins, including beclin-1, autophagy-related gene 7 (ATG7), and ATG12-ATG5 complex (23, 24, 45).

Collectively, these findings now demonstrate how Survivin serves as a critical signaling node in the ability of oncogenic KRAS to regulate MYC expression, by both preventing it from being dephosphorylated at a key regulatory site and by inhibiting a specific degradative process (i.e., autophagy). When taken together with our earlier work showing that Survivin is an essential cargo for the actions of exosomes shed by both aggressive breast and pancreatic cancer cells, they highlight how this RAS signaling target can significantly impact the proliferation and survival of tumor cells as well as the overall tumor microenvironment (22, 46). Moreover, they further suggest that targeting Survivin can potentially be used as part of a strategy to treat PDAC, especially since increases in MYC expression is correlated with drug resistance in PDAC (43, 47, 48), coupled with the fact there are currently no clinically available drugs that directly target MYC (49, 50). YM155 has been shown to be well tolerated by cancer patients in phase 1 clinical trials (51, 52). Therefore, it will be of great interest to determine whether YM155 can be used in combination with other drugs as an approach to more effectively treat PDAC.

## Experimental procedures

### Cell culture, transfection, and treatments

All cells were maintained in an incubator with 5% CO_2_ at 37 °C. The mouse AK192, AK12282, and AK14837 PDAC cell lines were generated as previously described and generously provided to us by Haoqiang Ying (MD Anderson) (53). These cells were cultured in RPMI-1640 (Gibco) containing 10% fetal bovine serum (Gibco), and 1 μg/ml doxycycline (Millipore) was added to the medium to induce KRAS^G12D^ expression. The human PANC-1 and MIA PaCa-2 PDAC cell lines, as well as the MEFs, were grown in Dulbecco’s modified Eagle’s medium (Gibco) containing 10% fetal bovine serum. pCDNA3 expression constructs encoding Myc-tagged Survivin (Sino Biological), HA-tagged MYC (Addgene #74164) (54), V5-tagged CIP2A (Addgene #119287) (55), GFP-tagged MYC (Addgene #42142) (56), and mCherry-LC3B (Addgene #40827) (57) were transfected into MEFs using PEI (Sigma) and PDAC cells using FuGENE 6 (Promega) per the manufacturer’s instructions. To knockdown Survivin expression in mouse cell lines, an siRNA-targeting mouse Survivin was used (Thermo Fisher Scientific, #S62463), while for the knockdown of Survivin in human cell lines, siRNAs targeting human Survivin were used (Horizon, #J-003459-08 and #J-003459-09). A scrambled negative control siRNA (Thermo Fisher Scientific, #4390844) was used in all experiments involving siRNAs and were introduced into cells using Lipofectamine RNAiMAX (Thermo Fisher Scientific). Cells were treated with the indicated concentrations of YM155 (Selleckchem), chloroquine (Sigma), 10074-G5 (Sigma), Sotorasib (AMG-510, MedChemExpress), MG-132 (Cayman Chemicals), SCH772984 (Selleckchem), or an equivalent amount of DMSO (Sigma).

### Western blot analysis

Cells were lysed using lysis buffer (25 mM Tris, 100 mM NaCl, 1.0 mM EDTA, 1 mM DTT, 1 mM NaVO_4_, 1 mM β-glycerol phosphate, 1% Triton X-100, 1 μg/ml aprotinin, and 1 μg/ml leupeptin) and quantified by the Bradford assay (Bio-Rad). The lysates were normalized based on protein concentrations, resolved on a 4 to 20% gradient SDS-PAGE gels (Invitrogen), and the proteins were transferred to PVDF membranes (Thermo Fisher Scientific). The membranes were blocked using 5 % bovine serum albumin (Sigma) dissolved in TBST buffer (19 mM Tris, 2.7 mM KCl, 140 mM NaCl, and 0.5 % Tween-20) before being incubated overnight at 4 °C with one of the following antibodies from Cell Signaling Technology which is diluted 1:1000 in TBST buffer: Caspase 3 (#9662), CIP2A (#14805), EGFR (#9267), phosphor-EGFR(Y1068, #2234), ERK (#4695), phospho-ERK1/2 (T202/Y204, #9101), HA (#3724), MYC (#5605), phospho-MYC (T58, #46650), phospho-MYC (S62, #13748), p62 (#39749), PP2A-A (#2041), PP2A-B (#2290), PP2A-C (#2259), RAS (#3965), Survivin (#2808), V5 (#13202), and Vinculin (#13901, and Sigma #V9131). The blots were then incubated with horseradish peroxidase–conjugated secondary antibodies (anti-rabbit IgG, Cell Signaling Technology #7074; anti-mouse IgG, Cell Signaling Technology #7046) for 1 h, washed with TBST buffer, incubated with Clarity Western ECL Substrate (Bio-Rad), and developed using HyBlot CL Autoradiography Film (Thomas Scientific) and a Konica Minolta SRX-101A developer, or imaged using a ChemiDoc MP (Bio-Rad).

### Quantitative PCR

The RNA isolated from cells treated with 250 nM YM155, or DMSO, for 24 h using the Direct-zol RNA miniprep kit (Zymo Research) was reverse transcribed to complementary DNA using Superscript III Reverse Transcriptase (Invitrogen) and oligo dT. qPCR was performed on the samples using a 384-well reaction plate (Thermo Fisher Scientific) and the ViiA 7 Real-Time PCR system (Thermo Fisher Scientific). The primers used to detect MYC transcripts (TTCGGGTAGTGGAAAACCAG, AGTAGAAATACGGCTGCACC), CIP2A transcripts (TCAGGACCCACGTTTGATTAC, GGCATTGTTTGCTGCTATACTT), and actin transcripts (CATGTACGTTGCTATCCAGGC, CTCCTTAATGTCACGCACGAT) were purchased from Integrated DNA Technology.

### Lentivirus transduction

Lentiviruses were generated by transfecting HEK293T cells with the packaging constructs pMD2.G (Addgene #12259) and pCMV delta R8.2 (Addgene #12263), as well as shCIP2A (KIAA1524, Sigma #TRCN0000274969) using FuGENE 6. The medium containing the viral particles was harvested after 24 and 48 h and combined with polybrene (10 μg/ml, Sigma) before being added to target cells.

### Fluorescence microscopy

Cells ectopically expressing the indicated constructs were seeded on glass cover slips and treated with YM155. The cells were fixed using 4% formaldehyde diluted in PBS. Hoechst stain (Invitrogen) was added to cells to label their nuclei, and the cover slips were mounted on glass slides and imaged using a Zeiss Observer.Z1 microscope with a 63× objective and an Axio-Cam HRc camera. The images were processed using ImageJ (ImageJ.org).

### Autophagic flux assay

Cells ectopically expressing the tandem tagged reporter construct mCherry-EGFP-LC3B were generated as described previously (58) and were used to label autophagosomes. The cells were cultured in glass-bottom petri dishes (MatTEK) and treated with YM155 or DMSO overnight. Live-cell imaging was performed on the samples using either a Zeiss LSM 700 or LSM 910 confocal laser scanning microscope with a 63× objective, a 488 nm excitation laser, and a 561 nm excitation laser. Due to the sensitivity of EGFP fluorescence to acidic environments, which occurs when autophagosomes fuse with lysosomes, it can be used to readout changes in the rates of autophagy-dependent degradation. ImageJ was used to determine the relative ratio of the mCherry *versus* EGFP signal, which is presented as autophagic flux (59). Additional information regarding how this assay is performed was described previously (58, 59).

### CCK-8 cell growth assay

Cells (1 x 10^4^) plated in each well of a 96-well dish were treated with culturing medium containing the indicated combinations and concentrations of YM155, CQ, 10074-G5, Sotorasib, or DMSO. After 48 h, 10 μl of CCK-8 (Dojindo) was added to each well and incubated for 30 min. The 96-well plate was analyzed by a SPARK Multimode Microplate Reader (Tecan) at an absorbance of 450 nm.

### Data analysis

All experiments were independently performed a minimum of three times. Quantitative data were analyzed using GraphPad Prism (graphpad.com) and presented as means ± standard errors. Statistical significance of the findings was determined using Student’s t-tests; ∗*p* < 0.05, ∗∗*p* < 0.01, ∗∗∗*p* < 0.001, and ∗∗∗∗*p*< 0.0001.

## Data availability

All data are contained within the text.

## Supporting information

This article contains supporting information.

## Conflict of interest

The authors declare that they have no conflicts of interest with the contents of this article.
