# Supplementary material for: Oncogenic RAS promotes MYC protein stability by upregulating the expression of the inhibitor of apoptosis protein family member Survivin
Source: J Biol Chem. 2022 Dec 26;299(2):102842. doi: 10.1016/j.jbc.2022.102842 (PMC9860443; doi:10.1016/j.jbc.2022.102842)
Supplement: Supporting Information [file mmc1.docx]

**Oncogenic RAS promotes MYC protein stability by upregulating the expression of the inhibitor of apoptosis protein (IAP) family member Survivin**

Wen-Hsuan Chang^1,2^, Yinzhe Liu^1^, Emma A. Hammes^1^, Kirsten L. Bryant^2,3^,
Richard A. Cerione^1,4*^, and Marc A. Antonyak^4*^

1- Department of Chemistry and Chemical Biology, Cornell University, Ithaca, NY 14853, USA

2- Lineberger Comprehensive Cancer Center, University of North Carolina at Chapel Hill, Chapel Hill, NC 27599, USA

3- Department of Pharmacology, University of North Carolina at Chapel Hill, Chapel Hill, NC 27599, USA

4- Department of Molecular Medicine, Cornell University, Ithaca, NY 14853, USA

*-Corresponding authors: MAA; [maa27@cornell.edu](mailto:maa27@cornell.edu) and RAC; rac1@cornell.edu

**Supplementary Figures**

**Figure S1**

Cell counting kit-8 (CCK-8) cell growth assays were performed on MIA PaCa-2 cells treated without (DMSO) or with Sotorasib. The data are presented as means ± standard errors of at least three experiments; ***P* < 0.01 by Student’s *t* test.

**
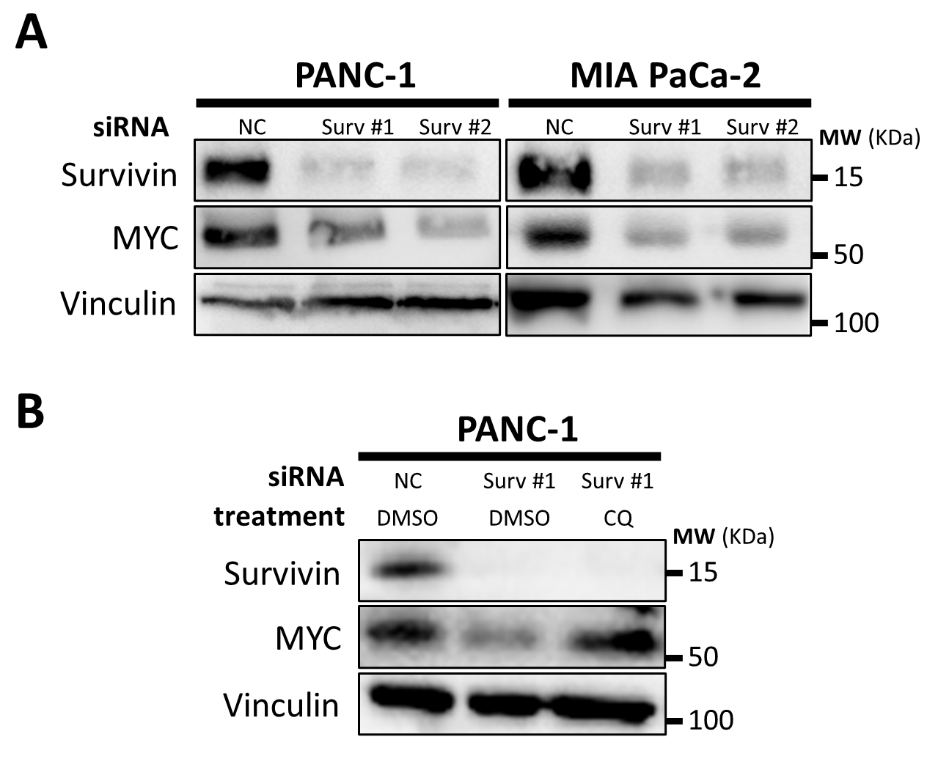
**

**Figure S2**

(**A**) Representative Western blot analysis of Survivin and MYC expression in PANC-1 and MIA PaCa-2 cells transfected with negative control (NC) or two different Survivin-targeting (Surv #1 and Surv #2) siRNAs. (**B**) Representative Western blot analysis of Survivin and MYC expression in PANC-1 and MIA PaCa-2 cells transfected with negative control (NC) or a Survivin-targeting (Surv #1) siRNA and treated without (DMSO) or with chloroquine (CQ). In (*A and B*), vinculin was used as the loading control.


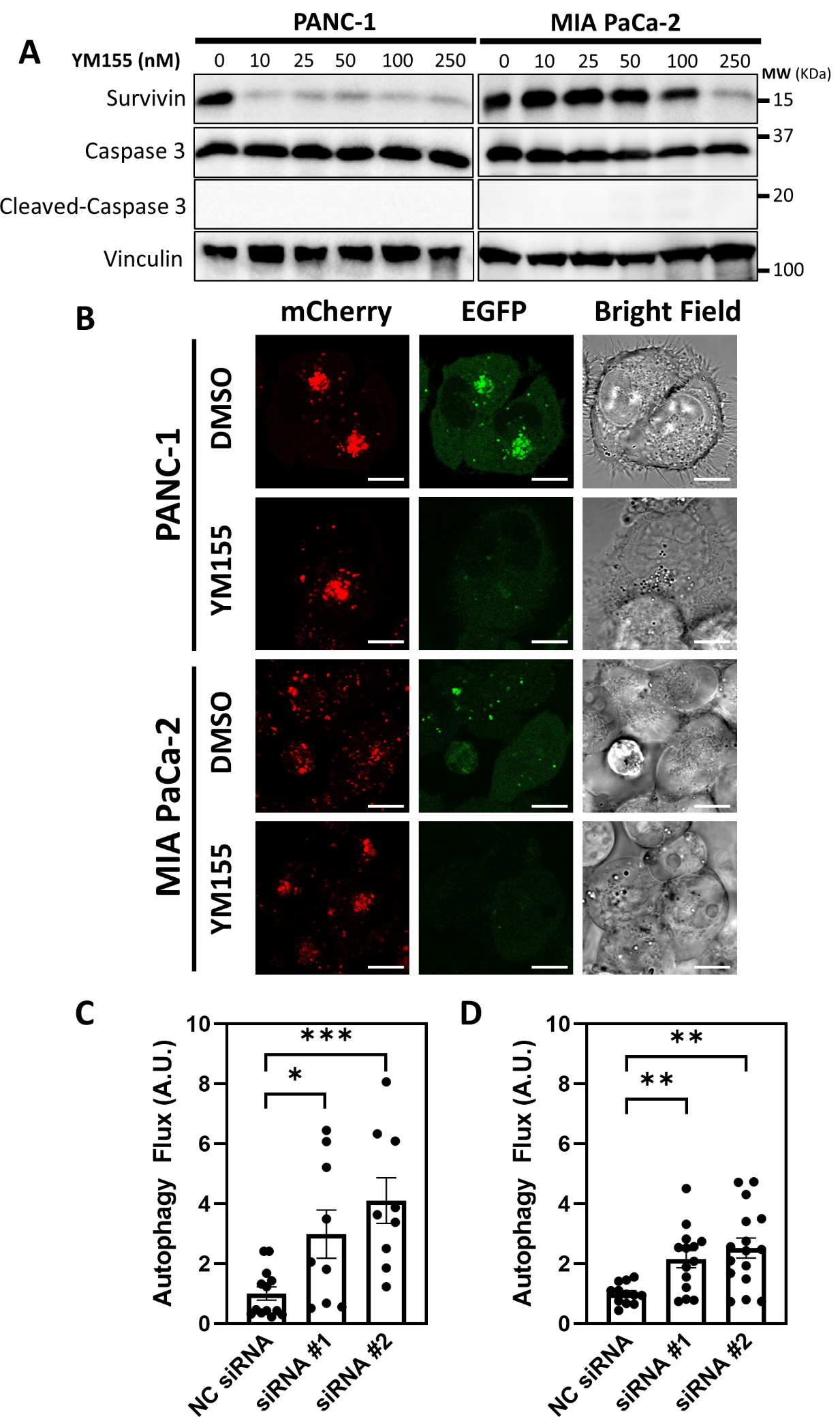


**Figure S3**

(**A**) Representative Western blot analysis of Survivin and caspase 3 expression in PANC-1 and MIA PaCa-2 cells treated with increasing concentrations of YM155 for 24 hours. Very little cleaved caspase 3, as an indicator of apoptosis, was detected in the cells treated with YM155. Vinculin was used as the loading control and this experiment was performed a minimum of three independent times, with similar results being obtained each time. (**B**) Representative fluorescence and bright field confocal microscopy images of PANC-1 and MIA PaCa-2 cells ectopically expressing mCherry-EGFP-LC3B treated without (DMSO) or with 250 nM YM155 for 24 hours. The scale bars represent 10 µm. (**C and D**) Autophagic flux assays were performed on (C) PANC-1 and (D) MIA PaCa-2 cells transfected with negative control (NC) or two different Survivin-targeting (Surv #1 and Surv #2) siRNAs. The data are presented as means ± standard errors of at least three experiments; ***P* < 0.05, ***P* < 0.01, and ****P* < 0.001 by Student’s *t* test.


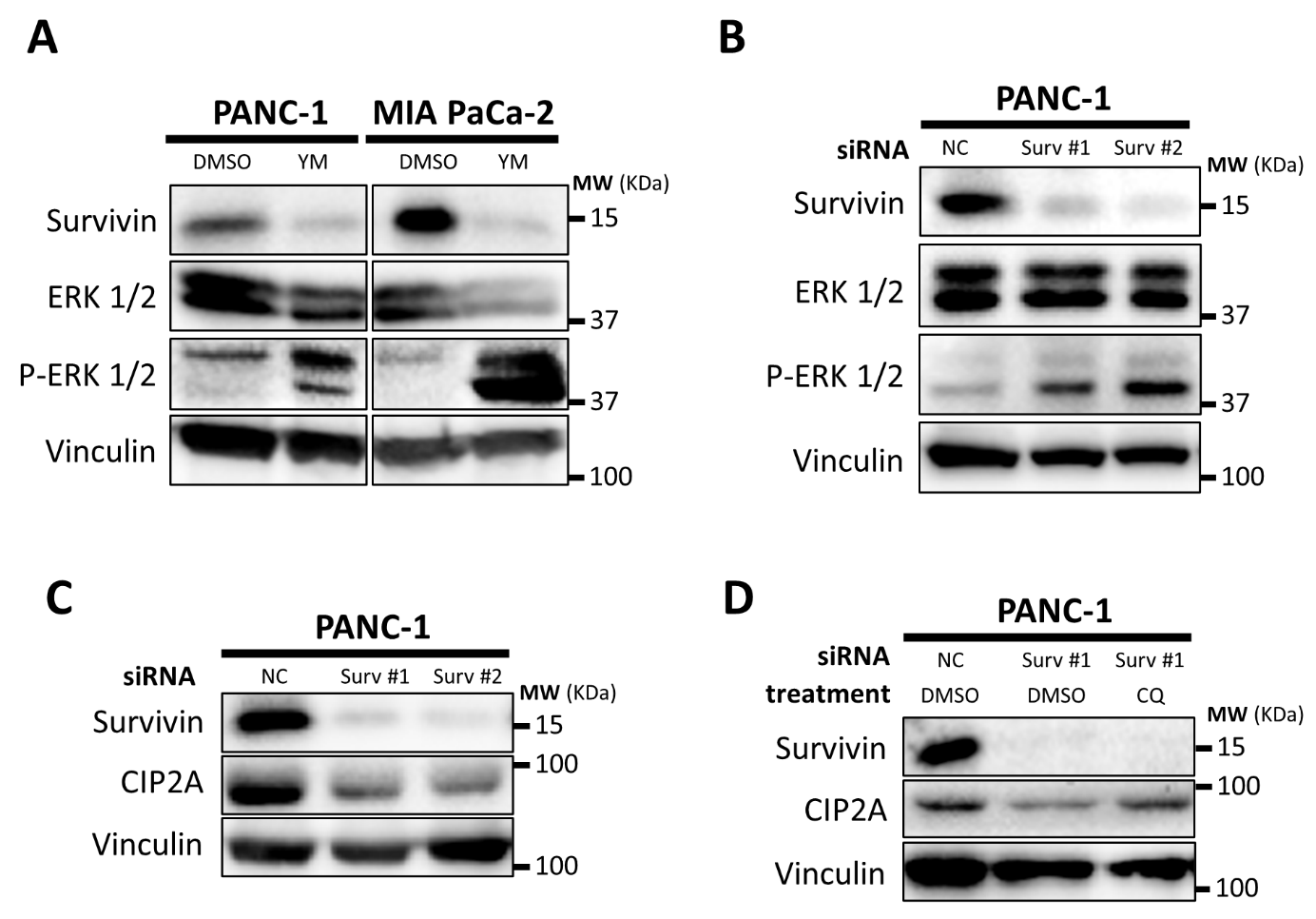


**Figure S4**

(**A**) Representative Western blot analysis of Survivin, ERK1/2, and phospho-ERK1/2 (P-ERK1/2) levels in PANC-1 and MIA PaCa-2 cells treated without (DMSO) or with 250 nM YM155 (YM) for 8 hours. (**B**) Representative Western blot analysis of Survivin, ERK1/2, and phospho-ERK1/2 (P-ERK1/2) levels in PANC-1 and MIA PaCa-2 cells transfected with negative control (NC) or two different Survivin-targeting (Surv #1 and Surv #2) siRNAs. (**C**) Representative Western blot analysis of Survivin and CIP2A expression in PANC-1 and MIA PaCa-2 cells transfected with negative control (NC) or two different Survivin-targeting (Surv #1 and Surv #2) siRNAs. (**D**) Representative Western blot analysis of Survivin and CIP2A expression in PANC-1 and MIA PaCa-2 cells transfected with negative control (NC) or a Survivin-targeting (Surv #1) siRNA and treated with DMSO or chloroquine (CQ). In (*A-D*), vinculin was used as the loading control.
